# Minocycline mitigates the effect of neonatal hypoxic insult on human brain organoids

**DOI:** 10.1038/s41419-019-1553-x

**Published:** 2019-04-11

**Authors:** Erin M. Boisvert, Robert E. Means, Michael Michaud, Joseph A. Madri, Samuel G. Katz

**Affiliations:** Department of Pathology, Yale University School of Medicine, New Haven, CT USA

## Abstract

Neonatal hypoxic injury (NHI) is a devastating cause of disease that affects >60% of babies born with a very low birth weight, resulting in significant morbidity and mortality, including life-long neurological consequences such as seizures, cerebral palsy, and intellectual disability. Hypoxic injury results in increased neuronal death, which disrupts normal brain development. Although animal model systems have been useful to study the effects of NHI, they do not fully represent the uniqueness and complexities of the human brain. To better understand the effects of hypoxia on human brain development, we have generated a brain organoid protocol and evaluated these cells over the course of 6 months. As anticipated, the expression of a forebrain marker, FOXG1, increased and then remained expressed over time, while there was a transition in the expression of the deep-layer (TBR1) and upper-layer (SATB2) cortical markers. In addition, ventral genes (Eng1 and Nkx2.1) as well as markers of specialized nonneuronal cells (Olig2 and GFAP) also increased at later time points. We next tested the development of our in vitro cerebral organoid model at different oxygen concentrations and found that hypoxia repressed gene markers for forebrain, oligodendrocytes, glial cells, and cortical layers, as well as genes important for the migration of cortical neurons. In contrast, ventral markers were either unaffected or even increased in expression with hypoxic insult. Interestingly, the negative effect of hypoxia on the dorsal brain genes as well as oligodendrocytes, and neuronal progenitors could be mitigated by the use of minocycline, an FDA-approved small molecule. Taken together, we have generated a unique and relevant in vitro human brain model system to study diseases such as NHI as well as their potential treatments. Using this system, we have shown the efficacy of minocycline for human NHI.

## Introduction

Chronic hypoxia prior to or around the time of birth caused by complications such as premature birth or neonatal hypoxic injury (NHI) is a common cause of neonatal death and neurodevelopmental handicaps^1,2^. In fact, in human neonates, NHI is the most frequent source of death and disability^3^. Improvements in care have led to an increase in viability after chronic hypoxia, especially in developed countries. However, there has been a subsequent increase in the number of survivors who often have life-long neurodevelopmental handicaps as a consequence of hypoxic insult such as cerebral palsy, seizures, epilepsy, and cognitive impairment^3–8^.

The lack of an accurate model system has been one of the biggest challenges in studying diseases of the human brain. In addition to the plethora of knowledge that we have gained using human pluripotent stem cells in 2D neuronal systems, recent work in 3D brain organoids also allow for the evaluation of cellular interactions and networks^9,10^. This system augments the knowledge gained through the use of animal models and postmortem tissue to uncover more about the development of the human brain in a unique way. It has been demonstrated that brain organoids have similar cell types as well as gene expression and epigenetic profiles to human fetal tissue^11–13^. Thus, brain organoids are a particularly good way to study the neonatal brain. The protocol for the model system utilized in this work was derived from a combination of existing protocols^14–17^.

Minocycline is a second-generation semi-synthetic tetracycline derivative that has many noncanonical functions, including various neuroprotective properties^18,19^. It is the most lipophilic of the tetracycline family members, which allows it to easily cross the blood–brain barrier^20,21^. Minocycline has been utilized for over 50 years for common conditions such as acne, such that there is a vast quantity of long-term data which indicates a very low frequency of serious adverse side effects^21–23^. In both mouse and human studies minocycline has been shown to be effective in many brain diseases^24,25^. Mouse studies have shown efficacy in using minocycline for treating the effects of chronic neonatal hypoxia, but it has not yet been tested in humans^1,26–29^. In this study, we used brain organoids in a hypoxia chamber as a means to model human neonatal hypoxic stress. We found that minocycline is very effective in blocking the negative effects of hypoxic insult that occur, particularly in the cortical brain regions.

## Results

**Fig. 1:**
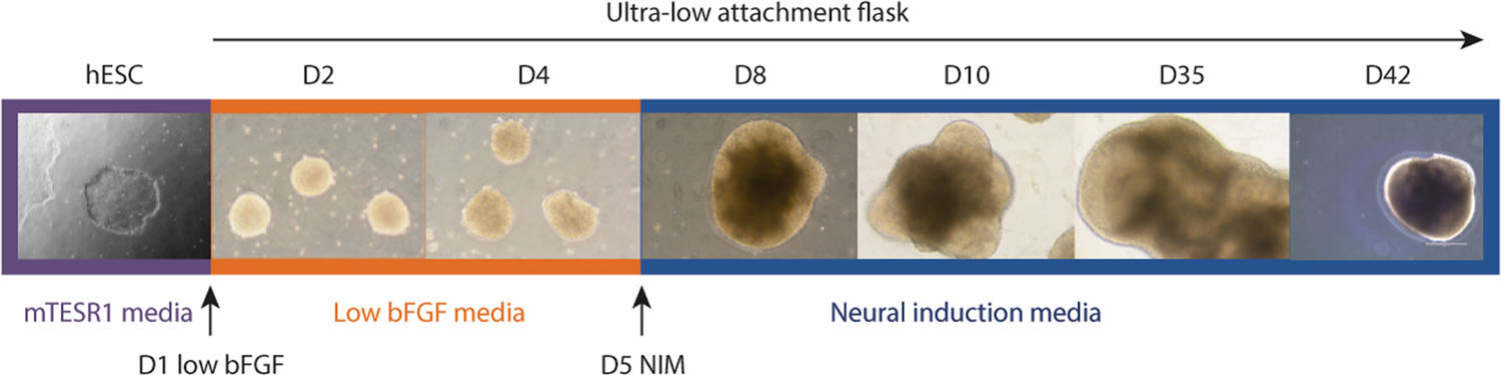
A schematic diagram of the overall protocol with representative images. The H9 hESCs were removed from the substrate and allowed to form spheres within a T75 ultra-low attachment flask (purple box). The cells were weaned off of the bFGF (orange box) until day 5 where they were first exposed to neural induction media (blue box). D, day

A self-directed organization method was employed for the generation of the brain organoids for this study. The H9 human embryonic stem cells (hESCs) (Fig. 1, purple box) were removed from the substrate using dispase, gently broken into small pieces, and allowed to form spheres by placing them into a T75 ultra-low attachment flask. This led to the formation of bright and healthy embryoid bodies (EBs). Staring at day 1 (Fig. 1, orange box), the EBs were weaned off of the bFGF by replacing the mTESR media with non-bFGF-containing media containing a bFGF supplement that was decreased daily (Fig. 1). On day 5 (Fig. 1, blue box), the cells were switched to a neural induction media (NIM) in order to induce the cells to become neuronal. By day 8 neural rosettes were observed, by day 10 neural budding was evident, and by day 35 there was a vast expansion of the neural rosettes (Fig. 1 and Supplementary Fig. 1). These organoids were allowed to grow in culture for up to 6 months, and samples were harvested at various time points for histology (H&E), immunofluorescence (IF), or quantitative reverse transcription PCR (qPCR).

**Fig. 2:**
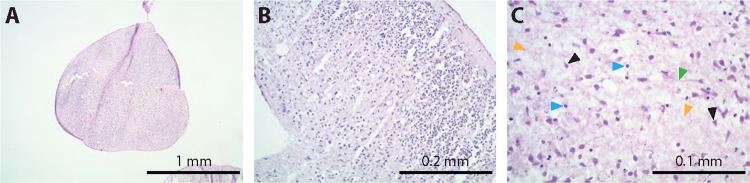
Histology of 5-month-old organoids. The organoids shared similarities to human brain tissue. There was no obvious evidence of necrosis by histology even after 5 months of culture (**a**). The brain organoids show some cortical-like organization (**b**). Various types of cell morphologies reminiscent of neurons (black arrow heads) and glia (blue arrow heads) can be observed (**c**). There was also the formation of synapses (green arrow head) and neuropil (orange arrow heads) (**c**)

**Fig. 3:**
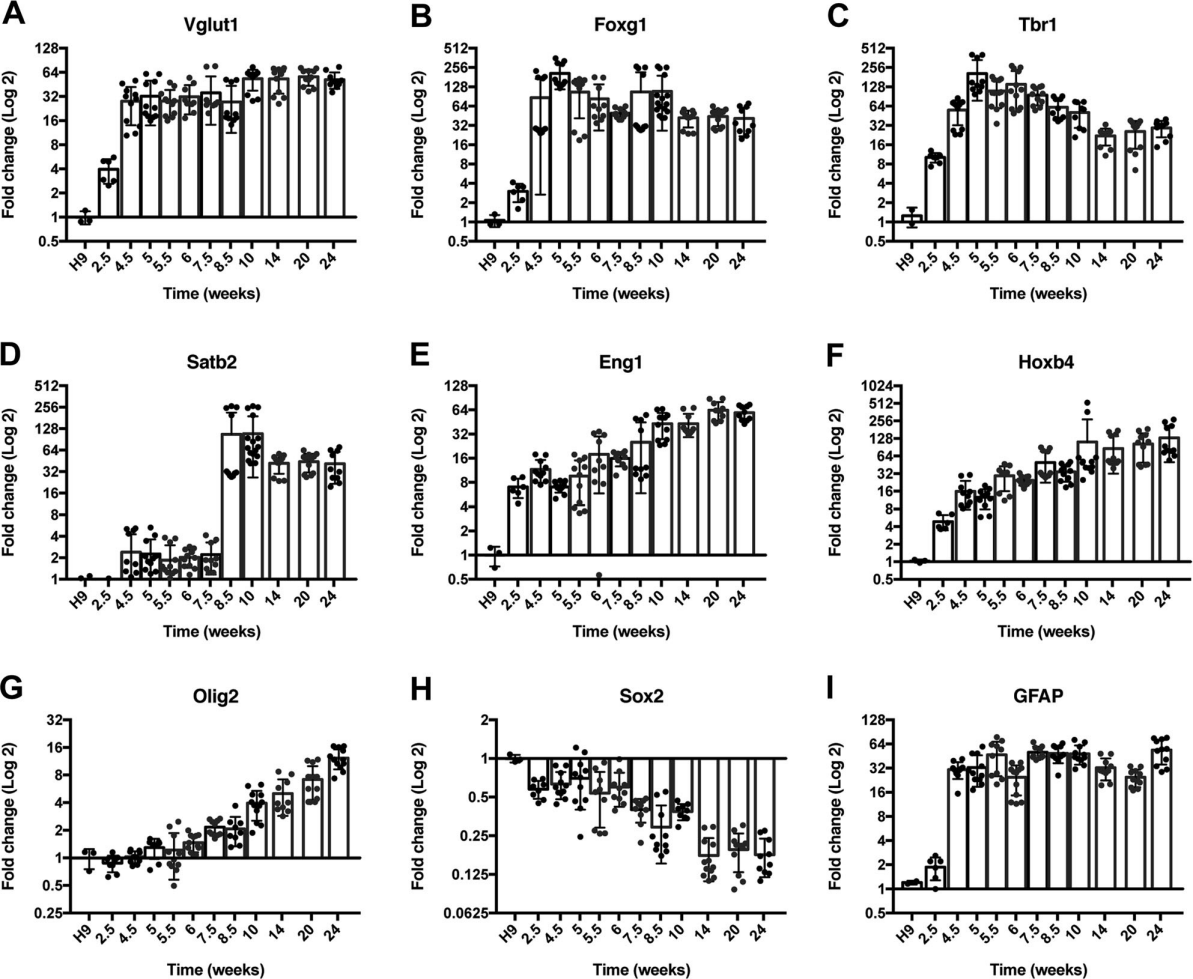
Time course of expression of key developmental genes. The glutamate marker Vglut1 (**a**) was expressed early on and remained highly expressed over the course of 6 months (*p* < 0.0001). While the expression of the forebrain marker Foxg1 (**b**) was relatively consistent (*p* < 0.0001), the expression of cortical layer markers (**c**, **d**) changed appropriately with time (Tbr1, *p* < 0.0001; Satb2, *p* < 0.0001). The expression of more ventral markers increased over time (**e**, **f**) (Eng1, *p* < 0.0001; Hoxb4, *p* < 0.001). The oligodendrocyte marker Olig2 (**g**) increased over time (*p* < 0.0001). The expression of Sox2 (**h**) decreased over time (*p* < 0.0001). The expression of the radial glial/astrocyte marker GFAP (**i**) had some fluctuations but was relatively consistent over time (*p* < 0.0001). Fold change was relative to the H9 hESC starting line. For each time point, a minimum of three separate samples were harvested from different batches of cells. Each of these samples were then run in triplicate. Statistical analysis (one-way ANOVA) was performed using Prism software. GAPDH values are in Supplementary Fig. 12A

After 5 months in culture, H&E-stained sections indicated that the organoids shared similarities to human brain tissue with no obvious morphological signs of necrosis by histology (Fig. 2a) and very few positive cells by immunostaining with cleaved caspase 3 and pMLKL (Supplementary Fig. 2). Interestingly, the brain organoids showed some cortical-like organization and evidence of layering (Fig. 2b). Various types of cells could be seen in the slices with histological morphology that resembled neurons (black arrow heads) and glia (blue arrow heads) as well as nicely spaced neuropil (orange arrow heads) (Fig. 2c). Even cells with Cajal–Retzius-like morphology were seen (Supplementary Fig. 3). In addition, there were evident synapses (green arrow head) that correlated with the qPCR data, which indicated that the glutamate marker Vglut1 (Fig. 3a) was increased from 2.5 to 4.5 weeks and then remained highly expressed over the course of 6 months.

A time course of key developmental genes over time was performed to determine if our model followed known developmental cues. The forebrain marker Foxg1 (Fig. 3b) was expressed as early as 2.5 weeks, increased at 4.5 weeks, and then remained expressed over time (*p* < 0.0001). It has been established that in humans, the deep-layer cortex forms before the upper-layer cortex, which is consistent with our data. The deep-layer cortical marker Tbr1 (Fig. 3c) increases up until 5 weeks and then decreases over time with a low at around 14 weeks (*p* < 0.0001), whereas the upper-layer cortical marker Satb2 (Fig. 3d) increases over time with the largest rise between 7.5 and 8.5 weeks (*p* < 0.0001).

Over the development of the organoid there was an increase in the expression of more ventral markers. More specifically, the expression of the midbrain/hindbrain gene Eng1 (Fig. 3e, *p* < 0.0001) and the hindbrain/spinal cord marker Hoxb4 (Fig. 3f, *p* < 0.001) showed a steady incline with time. In parallel with the ventral markers the oligodendrocyte marker Olig2 (Fig. 3g) also increased (*p* < 0.0001).

**Fig. 4:**
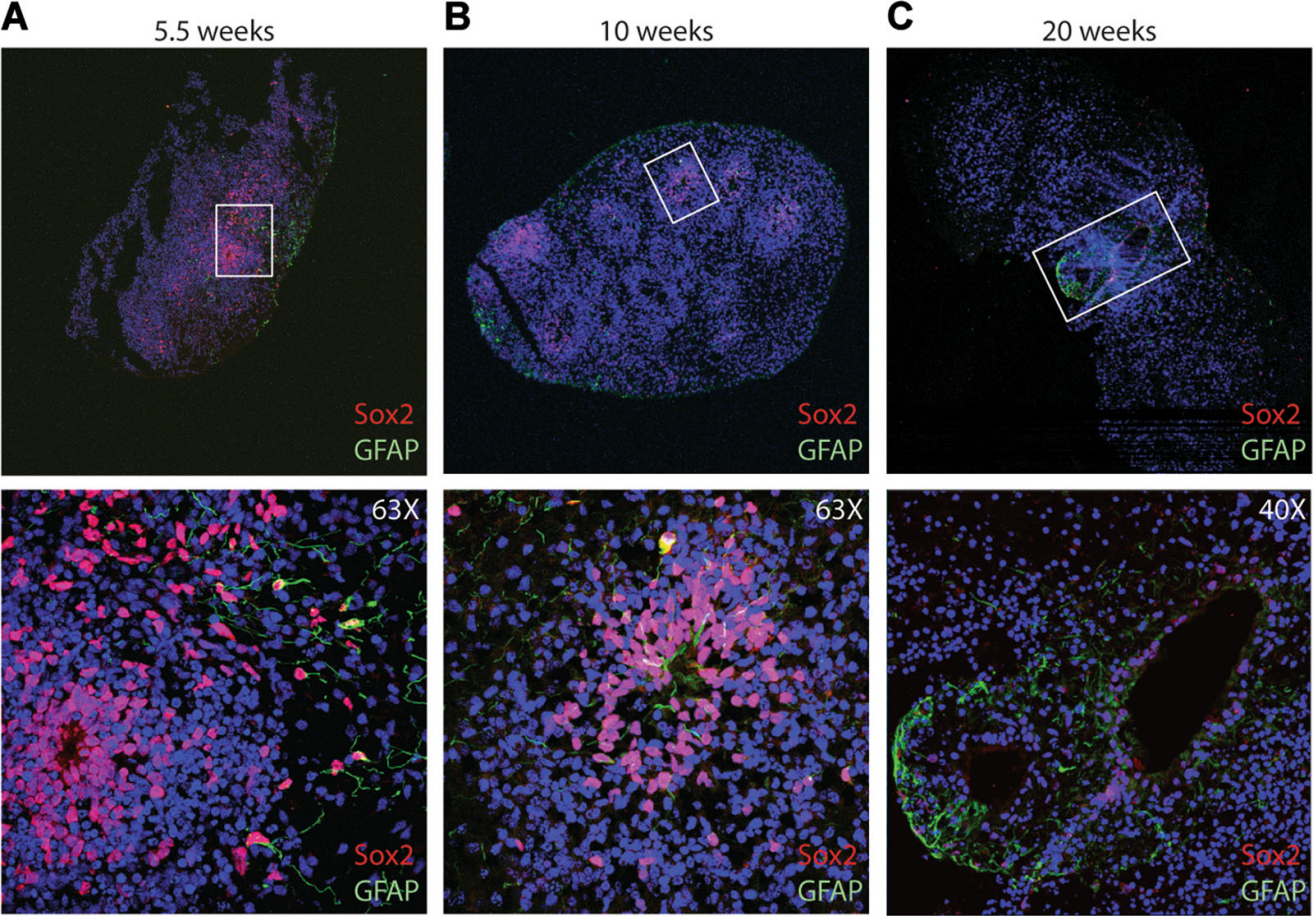
The Sox2 expression decreases over time and shows partial regionalization. Consistent with the qPCR data, there is a decrease in the protein expression of Sox2 by immunofluorescence as the cells mature, which can be seen at 5.5 weeks (**a**), 10 weeks (**b**), and 20 weeks (**c**) in culture

As expected, the expression of the stemness marker Sox2 (Fig. 3h) decreased over time (*p* < 0.0001), but never completely went away, as similar to the human brain, there are areas of progenitors within the organoids. In addition, the expression of the radial glial/astrocyte marker GFAP (Fig. 3i) had some fluctuations, but was relatively consistent over time after 4.5 weeks (*p* < 0.0001). The Sox2 and GFAP-positive areas are also seen via immunofluorescent staining (Fig. 4).

Consistent with the qPCR data, there is a decrease in the overall expression of Sox2 as the cells mature, which can be seen at 5.5 weeks (Fig. 4a), 10 weeks (Fig. 4b), and 20 weeks (Fig. 4c) in culture. In addition to the change in the overall expression, there was also change in the regionalization of the Sox2 expression as it became more refined between 5.5 and 20 weeks in culture (Fig. 4a–c, Supplementary Fig. 4). Likewise, the early neuronal marker, Pax6, appeared to decrease with time (Supplementary Fig. 5). There was an apparent trend toward an increase in the upper-layer cortical marker Satb2 and a decrease in the deep-layer cortical marker Ctip2 between 10 and 20 weeks (Supplementary Fig. 6). Similar to Sox2, FoxG1 and GFAP appeared to exhibit partial regionalization at 20 weeks (Supplementary Fig. 7).

Several other cell types were also present by immunofluorescent staining. Interestingly, other groups have shown that the human outer radial glial cell (oRGC) marker, HopX can be found in brain organoids^30^. Our human brain organoids also had positive expression of HopX, at each of the three time points evaluated (Supplementary Fig. 8). Our brain organoids also had some cells positive for the mature astrocyte marker, S100B (Supplementary Fig. 9) as well as the microglial marker, Iba1 (Supplementary Fig. 10).

**Fig. 5:**
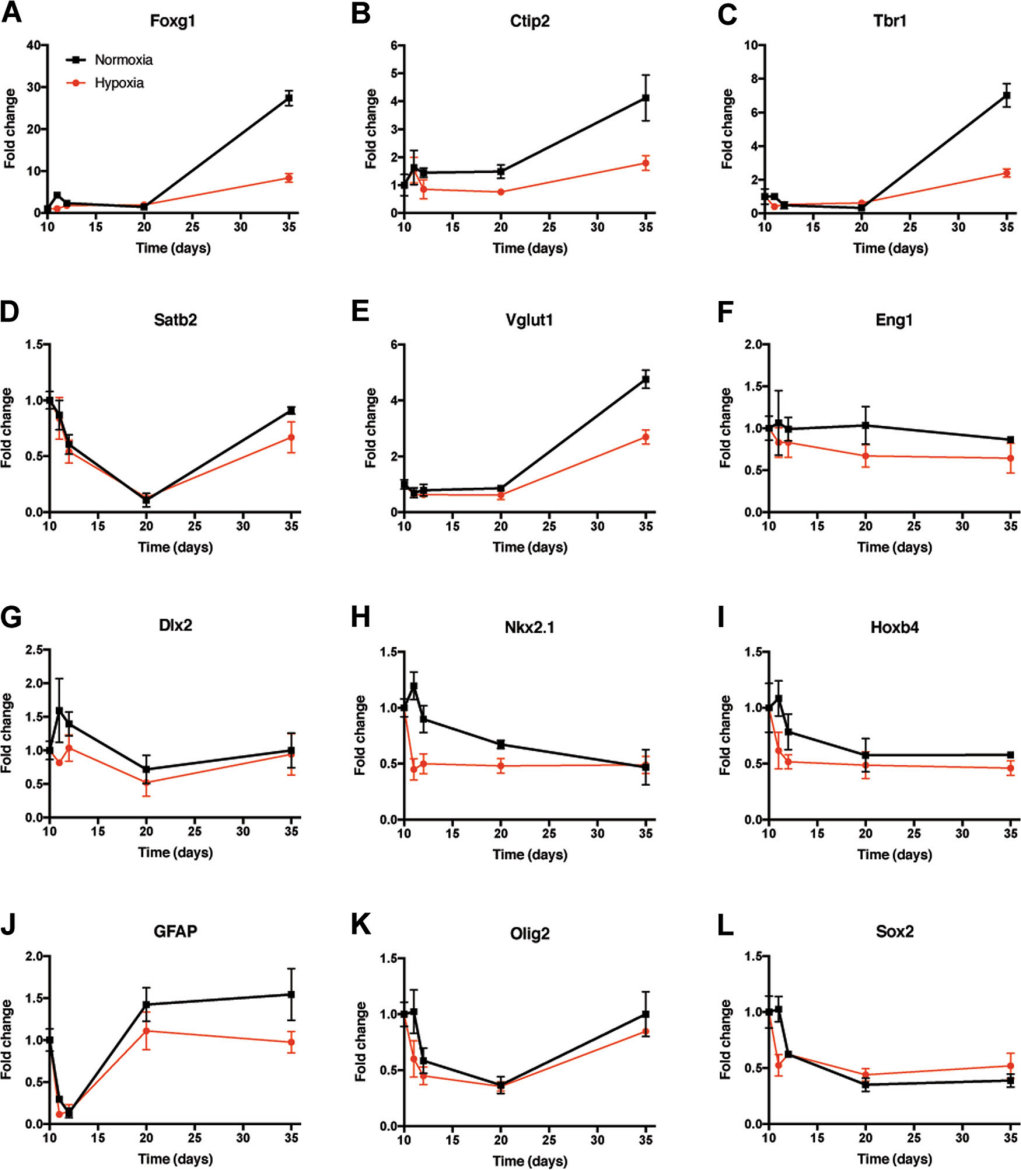
Hypoxia (8% oxygen) decreased the expression of forebrain markers compared to normoxia. Ten-day-old cerebral organoids placed into hypoxia (8% oxygen) revealed that cortical markers such as Foxg1 (**a**), Ctip2 (**b**), and Tbr1 (**c**) did not increase similar to as in organoids grown in normoxia (all *p* < 0.0001). Satb2 (**d**) also trended toward a similar pattern. Vglut1 (**e**) also increased more under normoxia than hypoxia (*p* < 0.0001 at day 35). More ventral markers such as Eng1 (**f**), Dlx2 (**g**), Nkx2.1 (**h**), and Hoxb4 (**i**) did not increase under normoxia or hypoxia (*p* = non-significant for days 20 and 35 for all three). GFAP (**j**) showed differences (*p* < 0.05) at days 20 and 35, whereas Olig2 (**k**) and Sox2 (**l**), showed differences on day 11. Each of these samples were run in triplicate. Statistical analysis (two-way ANOVA) was performed using the Prism software. GAPDH values are in Supplementary Fig. 12B

To test the effect of hypoxic stress, 10-day-old brain organoids were placed at 8% oxygen for 25 days and a time course of gene expression was performed to a final time point of day 35. During this time the normal increase in the expression of cortical markers was blunted in hypoxia (Fig. 5a–d). In particular, the expression of more dorsal cortical markers such as Foxg1 (Fig. 5a), Ctip2 (Fig. 5b), and Tbr1 (Fig. 5c) did not increase as much during hypoxia as during normoxia (*p* < 0.0001). Vlgut1 (Fig. 5e), a glutamate transporter abundant in the cortex, showed a similar trend (*p* < 0.0001). In contrast, more ventral genes such as Eng1 (Fig. 5f), Dlx2 (Fig. 5g), Nkx2.1 (Fig. 5h), and Hoxb4 (Fig. 5i) showed minimal decreases in expression under hypoxia early on, with no differences at later time points. Overall *p* values by two-way ANOVA are 0.004 for Eng1, 0.001 for Dlx2, <0.0001 for Nkx2.1, and 0.0007 for Hoxb4, but are not significant for any of the more ventral genes at the latter two time points. The astrocyte marker GFAP (Fig. 5j) and the oligodendrocyte marker Olig2 (Fig. 5k) exhibited mild, but statistically significant differences between normoxia and hypoxia (*p* < 0.01), which was more pronounced for later time points for GFAP and earlier time points for Olig2. The stem cell marker Sox2 (Fig. 5l) did not display statistically significant difference at 8% hypoxia overall, but did at day 11 (1 day after initiating hypoxia).

**Fig. 6:**
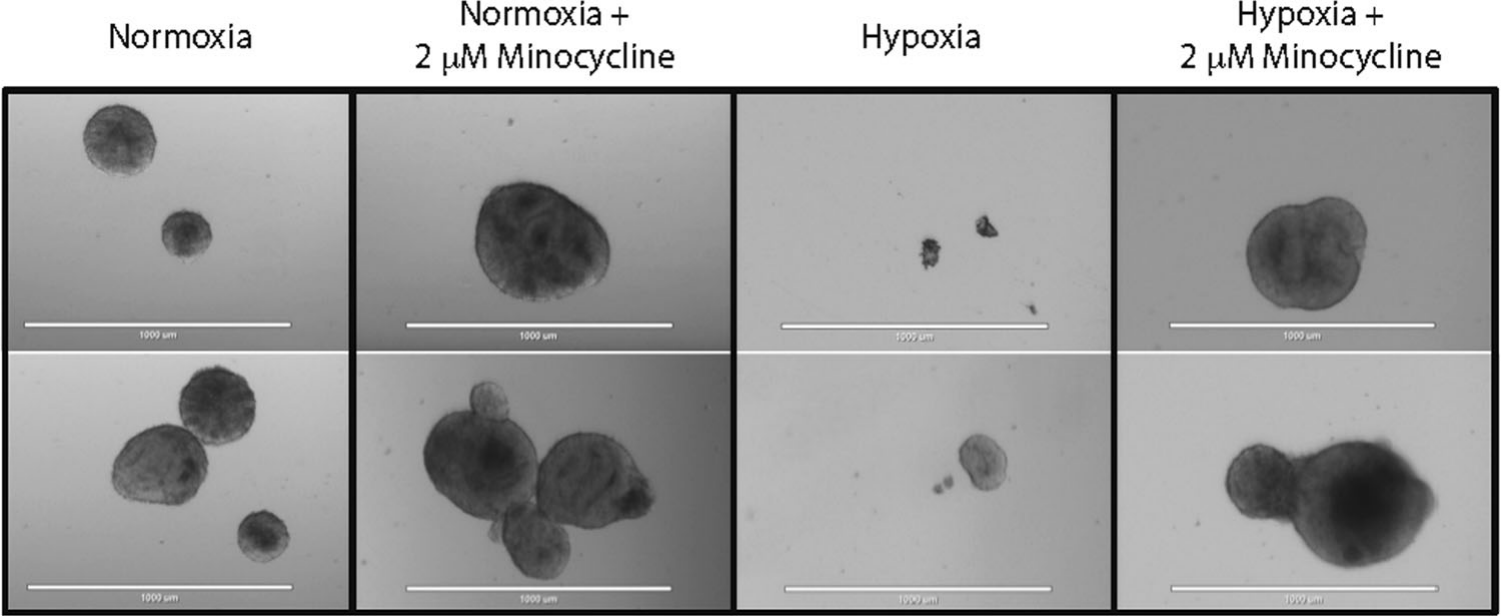
Minocycline rescued the morphological changes caused by hypoxic stress. Bright field images were taken of 10-day-old brain organoids grown in normoxia and hypoxia (1% oxygen), both without and with minocycline for 72 h. The experiment was repeated three separate times and samples from each of the groups were harvested. Scale bar = 1000 μm

**Fig. 7:**
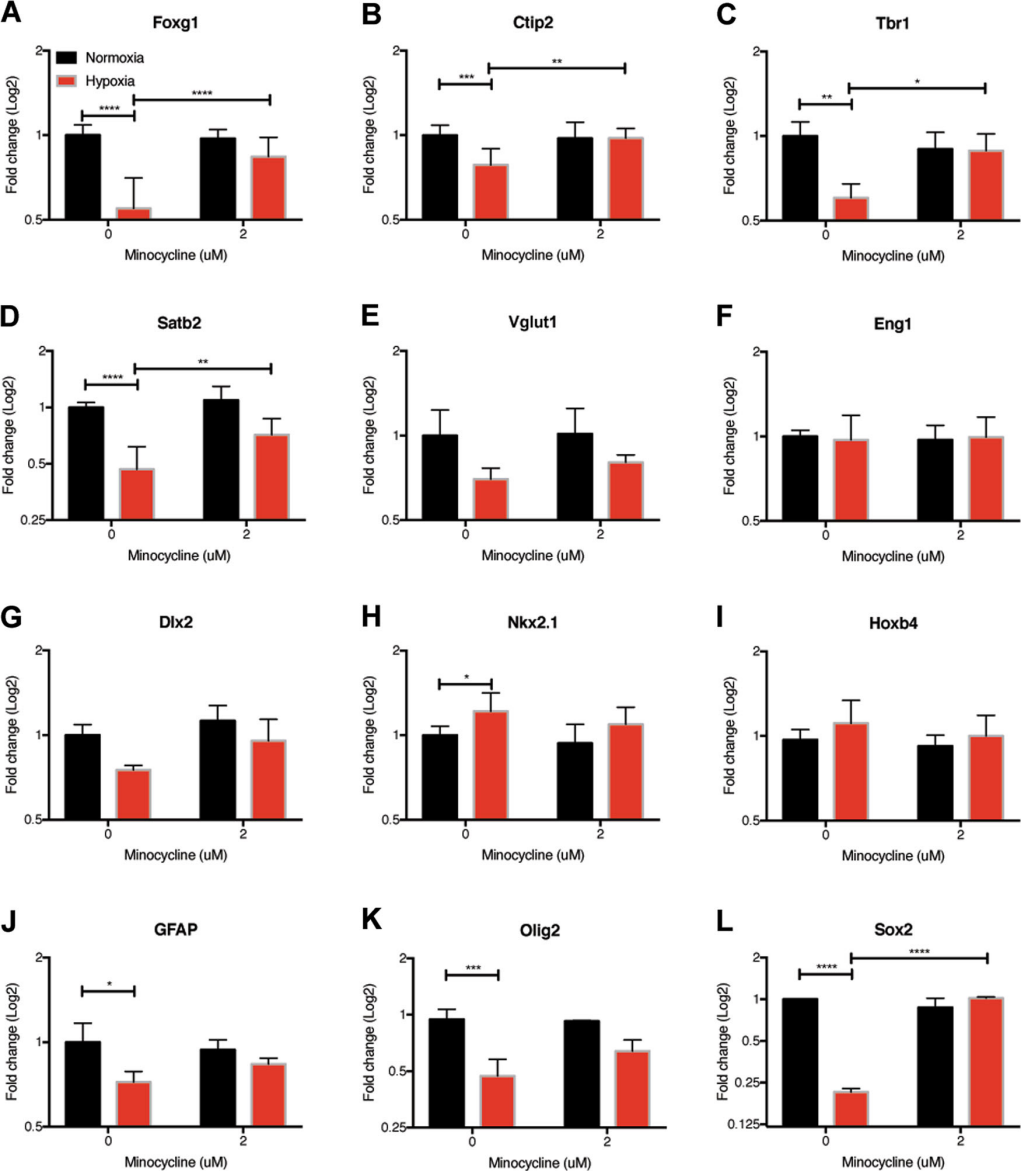
Changes in gene expression due to hypoxic stress can be protected by minocycline. The expression of cortical markers was significantly decreased in 10-day-old organoids grown in 1% oxygen for 72 h, but not in the presence of 2 μM minocycline (**a**–**d**). There was no decrease in Vglut1 (**e**) or ventral marker expression under hypoxic stress with or without minocycline (**f**–**i**). Hypoxia decreased the expression of GFAP (**j**), Oligo2 (**k**) and Sox2 (**l**), while minocycline rescued Sox2 expression. The experiment was repeated three separate times and samples from each of the groups were harvested. Each of these samples were then run in triplicate using qPCR. Statistical analysis (two-way ANOVA with multiple comparisons) was performed using Prism software (****p < 0.0001, ***p < 0.001, **p < 0.01, *p < 0.05). GAPDH values are in Supplementary Fig. 12C

As a second test of hypoxic stress, another cohort of studies were performed under 1% oxygen for 72 h and both the morphologic changes (Fig. 6) and the changes in gene expression (Fig. 7) were evaluated. Because minocycline has been shown to prevent damage due to lack of oxygen in mouse models^29^, we tested it using this human brain organoid model under normoxic and hypoxic conditions. After a dose curve was performed, 2 μM was chosen as the dose for subsequent experiments. Bright field images were taken under normoxia, and hypoxia, both without and with minocycline (Fig. 6). After 72 h in 1% oxygen, the organoids were mostly atrophied with only a few approaching the appearance of the organoids in 21% oxygen. When the minocycline was added to either the normoxic or hypoxic cultures the organoids appeared similar to the untreated, normoxic organoids.

The expression levels of critical neurodevelopmental genes were then evaluated in the samples that were exposed to 1% oxygen for 72 h both with and without the addition of minocycline. Similar to what was seen in the 8% oxygen study, the expression of cortical markers was significantly decreased under hypoxic stress (Fig. 7a–d, Supplementary Fig. 11A). Interestingly, these changes were protected by the addition of 2 μM minocycline. This effect was not cortical layer dependent as the expression of both deep-layer markers such as Ctip2 (Fig. 7b) and upper-layer markers such as Satb2 (Fig. 7d) were rescued by minocycline treatment. In stark contrast to what was observed in the dorsal markers, there was little change in the further ventral marker expression under hypoxic stress (Fig. 7f–i). The addition of minocycline did not significantly alter the expression of ventral markers (Fig. 7f–i). GFAP (Fig. 7j), Olig2 (Fig. 7k), and Sox2 (Fig. 7l) were all decreased under hypoxic stress and only Sox2 exhibited a statistically significant rescue by minocycline.

**Fig. 8:**
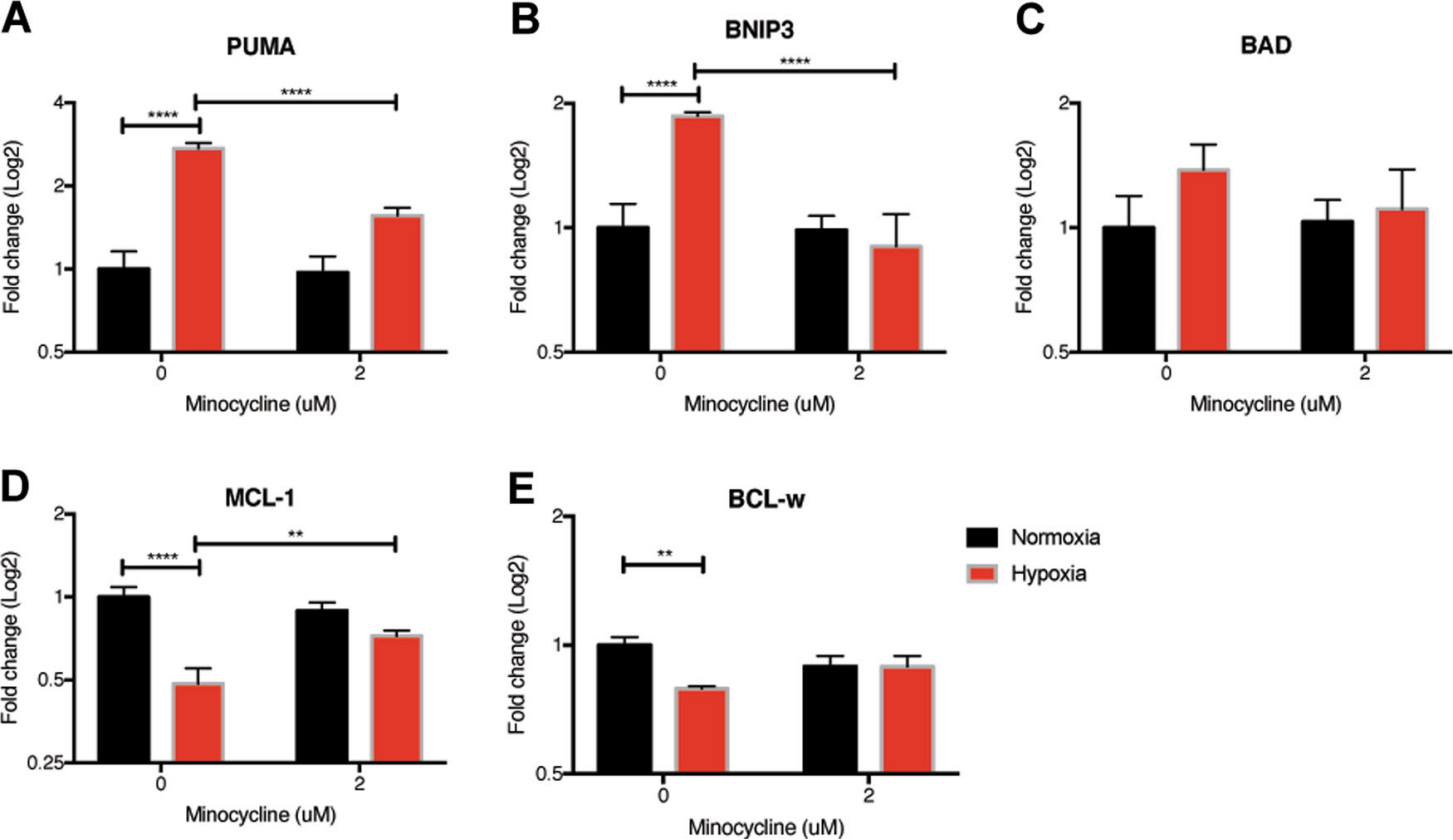
Changes in BCL-2 family member gene expression due to hypoxic stress can be protected by minocycline. The expression of proapoptotic proteins increased in 10-day-old organoids grown in 1% oxygen for 72 h, but not in the presence of 2 μM minocycline (**a**–**c**). The expression of antiapoptotic proteins decreased in 10-day-old organoids grown in 1% oxygen for 72 h, but not in the presence of 2 μM minocycline (**d**, **e**). Samples were processed similar to Fig. 7

Interestingly, the expression levels of Yap and Sox10, two transcription factors that modulate expression of multiple genes with neural functions similar to findings in mice^29^, were also significantly decreased in the organoids under hypoxic stress and rescued by minocycline (Supplementary Fig. 11B, C). In addition, proapoptotic BCL-2 family members, PUMA and BNIP3, increased under hypoxia, while antiapoptotic family members, MCL-1 and BCL-w, decreased (Fig. 8). Minocycline partially restored the expression levels of PUMA, BNIP3, and MCL-1 to normoxia levels.

## Discussion

In recent years there has been remarkable progress in the development of multiple human organoid systems. In these models, the development and interaction of multiple cell types create a rich milieu that mimics several aspects of the human tissue. Human brain organoids are particularly useful to study a hitherto relatively inaccessible and notably complicated organ.

Here, we have generated a simple and robust brain organoid system. Unlike most existing systems, we chose to avoid bioreactors, matrigel, and extrinsic signaling molecules to improve ease of use and cost effectiveness. Avoiding matrigel helped decrease the risk of contamination, lowered the cost, simplified imaging, and avoided the presence of unknown quantities of growth factors that copurify with the product during production. This simple system also enabled us to avoid the addition of exogenous growth factors. Thus, importantly, the changes that we saw in gene expression, including the increase in ventralization markers, and the markers of more mature cell types were due to innate factors within the brain organoids.

A second unique feature of our system relative to other brain organoid systems is the generation of the initial EBs using clumps of cells instead of single cells and letting them form spontaneously, which is more similar to the existing 2D neuronal differentiation protocols^14^. We rationalized that in addition to the lack of innate vasculature in existing models, being confined to the ultra-low attachment 96-well plates might also contribute to necrosis. Indeed, we did not observe any apparent issues with necrosis within our brain organoids for up to 5–6 months in culture when starting with EBs in T75 flasks. Several brain organoid systems have shown considerable variability between batches of brain organoids^10,15,31^. However, performing qPCR on organoids from at least three separate cultures yielded fairly consistent findings over multiple time points. As might be expected developmentally, and in agreement with other systems, we find a strong initial upregulation of the ventral forebrain marker FoxG1, followed by more dorsal genes at slightly later time points as seen at both the mRNA and protein levels. Given that neuronal differentiation from hESCs is known to generate mostly forebrain neurons, the subsequent expression of more ventral markers and oRGCs may suggest a more complete developmental program similar to that seen in the developing human CNS^32^.

Although human brain organoids offer many advantages as a model system, there are still some important caveats to the system, especially when studying diseases such as NHI. Chief among them, the lack of vasculature in existing brain organoids is a big limitation^10,33,34^. Blood flow and vascular cell-derived factors play significant roles in the response of the brain to hypoxic stress. For example, subsequent consequences of NHI such as intraventricular hemorrhage, would be better modeled with the presence of a vasculature. In addition, in other model systems it has been shown that minocycline can decrease the activation of microglia, which are one potential target of minocycline since it can decrease their activation^35–38^. Recently, other groups have made growth factor modifications to the existing brain organoid models and reported that it increases the number of microglia^39^. Thus, it would be interesting to observe the effect of minocycline on these organoids.

To our knowledge, this is the first study to show the effects of hypoxia on human brain organoids, and the potential therapeutic benefit of minocycline. In agreement with the prior mouse studies^40,41^, we find that hypoxia has a more profound effect on the forebrain cortex (e.g., Foxg1, Dcx1, Ctip2, and Satb2), relative to the more ventral brain regions (e.g., Nkx2.1, Eng1, and Hoxb4). A similar phenomenon has also been shown using mice exposed to hypoxic conditions where the hypoxia-regulated transcripts were downregulated in the forebrain, but upregulated in the hindbrain^41^. Since the more ventral (primitive) structures of the brain control vital life functions, it is not unreasonable to postulate that these regions are more protected from injury.

In prior work in the mouse, we have found that minocycline prevents the downregulation by hypoxia of a transcriptional program of neurogenesis controlled by YAP and Sox10^29^. In particular, these transcription factors regulate genes involved in proliferation, apoptosis, and neural development^26^. Physiologically, minocycline leads to improved cognitive outcome in hypoxic mice. In our brain organoid system, finding that minocycline mitigates the increase of proapoptotic PUMA and BNIP3 and the decrease of anti-apoptotic MCL-1, raises the possibility that minocycline may inhibit hypoxia-induced cell death. Thus, minocycline may rescue not only YAP and Sox10, but also their transcriptional programs in the hypoxic human brain organoids.

Although 2 μM minocycline essentially normalizes the expression of these key transcription factors in day 10 brain organoids at 1% oxygen for 72 h, there are countless future permutations that can be explored to determine the best use for minocycline in this human brain model. For example, hypoxia studies on older organoids could be interesting to evaluate the significance of treating at specific developmental time points. Varying the oxygen concentration to less severe levels might reveal even more dramatic effects of minocycline at lower doses. In addition, preconditioning the organoids to hypoxia would allow for the evaluation of genes important for neuronal protection in the human brain^42–44^. Our model system offers the opportunity to compare alternative therapies and pathways, like apoptosis, in a human context. Given the delicate nature of treating NHI patients at this critical point in their development, it is reassuring that our human brain organoid findings are in concert with prior mouse work and support the clinical development of minocycline for NHI.

## Materials and methods

### Stem cells

H9 hESCs were obtained through WiCell (Madison, WI, USA). These cells were maintained on a layer of growth factor reduced matrigel (Corning, Corning, NY, USA) in mTESR media (STEMCELL Technologies, Vancouver, Canada) according to the manufacturer’s instructions. At the hESC stage, the media were changed daily and the cells were weeded using glass tools as necessary.

### Differentiation protocol

On day 0 (D0), the medium was aspirated off and dispase (Thermo Fisher Scientific, Waltham, MA, USA) diluted in DMEM/F12 (Thermo Fisher Scientific) was added to the hESCs for ~14 min or until the edges of the cells rounded up but before they lifted off of the substrate. The dispase solution was removed and the cells were washed three times using DMEM/F12. Next, the cells were gently rinsed off of the substrate by pipetting. The cells from four standard density plates of hESCs were then placed into a single ultra-low attachment T75 flask (Corning) containing 30 mL of mTESR medium without bFGF. The next day, the flask was tilted so that the live cells were able to pool in the corner. Next, the majority of the media was aspirated off and replaced with 20 mL low bFGF media (DMEM/F12 supplemented with 1× N2, 1× B27, 1× l-glutamine, 1× NEAA, 0.05% BSA, and 0.1 mM MTG) supplemented with 30 ng/mL bFGF. On day 3, the medium was replaced with 20 mL of low bFGF media supplemented with 10 ng/mL bFGF. On day 5 the medium was replaced with neural induction media (NIM: DMEM/F12, 1X N2 supplement (N2 Neuroplex, Gemini Bio Products, West Sacramento, CA, USA), 0.1 mM MEM NEAA (Thermo Fisher Scientific), 2 μg/mL heparin (Sigma–Aldrich. St. Louis, MO, USA). After this point, the cells were maintained in NIM and half of the medium (~10 mL) was switched every other day. After 3 weeks in culture, the medium was also supplemented with Pen Strep (Thermo Fisher Scientific).

### Hypoxia protocol

An incubator was set at standard conditions (5% CO_2_, 37 °C) and hypoxia chamber (02 Control InVitro Cabinet, Coy Laboratory Products, Grass Lake, MI, USA) was placed within the incubator. The oxygen concentration within the chamber was carefully calibrated using both the oxygen sensor and a fyrite and set at 8% or 1% oxygen depending upon the experiment. For each experiment, the cells were differentiated following our protocol as described and at day 10, the cells were distributed evenly into T25 flasks (Corning) containing 8 mL of NIM. If minocycline (Sigma–Aldrich) was being added to the sample it was first re-suspended in PBS and added such that the final concentration was 2 μM. The samples that were not receiving minocycline were given the same amount of PBS and all of the samples were mixed well. The flasks were then placed into the incubator under standard conditions or within the hypoxia chamber.

### qRT-PCR

The samples were taken out of the flask and were gently spun in a 1.5-mL tube. The cells were rinsed with 1× DPBS three times and pelleted. The RNA was extracted from the cells using an RNeasy kit (Qiagen, Hilden, Germany), the OD values were measured using a NanoDrop (Thermo Fisher Scientific) and cDNA was made using the iScript cDNA Synthesis Kit (Bio-Rad, Hercules, CA, USA). Quantitative RT-PCR (qRT-PCR) was performed using a SYBR Green gene expression assay in a 20-μL mixture containing primers, cDNA, and iQ SYBR Green Supermix (Bio-Rad) and run on a qPCR machine (Bio-Rad, CFX96). First, using the standard curves, each set of primers was evaluated to ensure that only one amplicon was produced at the same rate as the housekeeping gene, GAPDH (Supplementary Fig. 12). For each experiment, the expression level of the mRNA was calculated using the ΔC_T_ method. Statistical analysis (*t*-test) was then done with the Prism 7 software. Primer sequences can be found in Supplemental materials.

### Immunohistochemistry

Brain organoids were taken out of culture at the appropriate time point, rinsed in PBS, and fixed in a 4% PFA (Electron Microscopy Sciences, Hatfield, PA, USA) solution at 4 °C for at least 2 days depending upon the size of the organoid. The organoids were then put through a sucrose gradient (10%, 20%, 30%) for 1 day at each concentration. Next, the organoids were embedded in OCT (Sakura Finetek. Torrance, CA, USA), sliced into 10 μM sections using a cryostat and placed onto slides (Thermo Fisher Scientific). The slides were washed three times with PBS, placed in a blocking solution (0.3% Triton X-100, 4% Normal Donkey serum (NDS, Jackson ImmunoResearch Laboratories Inc., West Grove, PA, USA) in PBS) for an hour, and placed at 4 °C overnight in a primary antibody solution (antibody, 0.1% Triton X-100, 4% NDS in PBS). The following day the slides were washed three times with PBS for 10 min each, and the secondary antibody was added for 1 h at room temperature. The slides were then washed three times with PBS for 10 min each. Next, a DAPI stain (Thermo Fisher Scientific, 1:10,000) was added for 5 min before performing three additional 10-min-long PBS washes. The primary antibodies included Ctip2 (1:100, Abcam, ab18465), Cleaved caspase 3 (1:400, Cell Signaling Technology, 9661, Danvers, MA), Foxg1 (1:200, Abcam, ab18259, Cambridge, UK), GFAP (1:1000, NeuroMab, N206A/8, Davis, CA), HopX (1:200, Santa Cruz Biotechnology, SC30216, Dallas, TX), Iba1 (1:500, Novus Biologicals, NB100-1028SS, Centennial, CO), Pax6 (1:350, Abcam, ab195045), pMLKL (1:250, Abcam, ab187091), Satb2 (1:100, Abcam, ab34735), Sox2 (1:300, MilliporeSigma, AB5603), The secondary antibodies included Alexa Fluor 488 goat anti-mouse (1:1000, Thermo Fisher Scientific, A11029), Alexa Fluor 488 donkey anti-rat (1:1000, Thermo Fisher Scientific, A21208), and Alexa Fluor 546 goat anti-rabbit (1:1000, Thermo Fisher Scientific, A11035).

### Image acquisition

Bright field images were taken using a digital camera attached to a Nikon TMS-F microscope or an EVOS cell imaging system. Fluorescent images were taken using a Leica (Wetzlar, Germany) SP5 confocal microscope, and the images were acquired using the LAS software. Z-stacks were compiled using the ImageJ software. Minor adjustments to the contrast or brightness were done for entire images and for each of the conditions/time points.

## Supplementary information

Supplementary information

Supplementary Figure 1

Supplementary Figure 2

Supplementary Figure 3

Supplementary Figure 4

Supplementary Figure 5

Supplementary Figure 6

Supplementary Figure 7

Supplementary Figure 8

Supplementary Figure 9

Supplementary Figure 10

Supplementary Figure 11

Supplementary Figure 12
