# Supplementary material for: Minocycline mitigates the effect of neonatal hypoxic insult on human brain organoids
Source: Cell Death Dis. 2019 Apr 11;10(4):325. doi: 10.1038/s41419-019-1553-x (PMC6459920; doi:10.1038/s41419-019-1553-x)
Supplement: Supplementary file 1 — Supplementary information [file 41419_2019_1553_MOESM1_ESM.docx]

Supplementary Information

Boisvert et al.

Minocycline mitigates the effect of neonatal hypoxic insult on human brain organoids.

**Supplementary Figure Legends**

**Supplementary Figure 1**. **Brightfield images of organoids**. Examples of cerebral organoids are shown from 4.5 to 20 weeks in culture showing growth by comparing size. The bar in the bottom right represents 1000 microns.

**Supplementary Figure 2**. **Minimal cleaved caspase 3 or phosphor-MLKL positive cells in organoids.** Representative examples of 10 week organoids stained with anti-p-MLKL (**A**) or 20 week organoids stained with anti-cleaved caspase 3 (**B**). Rare positive cells are indicated by arrowheads.

**Supplementary Figure 3**. **Cells with Cajal-Retzius morphology.** The arrowheads indicate examples of cells with Cajal-Retzius-like morphology located in the outermost portion of the organoid.

**Supplementary Figure 4. Single channel images corresponding to Figure 4.** DAPI (blue), Sox2 (red) and GFAP (green) are shown at 10X and 63X for 5.5 weeks (**A**), 10 weeks (**B**), and 20 weeks (**C**) in culture.

**Supplementary Figure 5. The expression of Pax6 decreases with maturation.** Pax6 (red) immunostaining in organoids at 5.5 weeks (**A**), 10 weeks (**B**), and 20 weeks (**C**) in culture.

**Supplementary Figure 6. Satb2 and Ctip2 expression.** Deep layer cortical marker Ctip2 and upper cortical layer marker Satb2 are expressed from 10 weeks (**A**) to 20 weeks (**B**) in culture.

**Supplementary Figure 7. Foxg1 and GFAP expression in organoids.** The expression of Foxg1 (red) and GFAP (green) became more regionalized from 10 weeks (**A**) to 20 weeks (**B**) in culture.

**Supplementary Figure 8. The expression of HopX is constant.**  HopX (red) immunostaining in organoids at 5.5 weeks (**A**), 10 weeks (**B**), and 20 weeks (**C**) in culture.

**Supplementary Figure 9. S100B expression in organoids.** S100B (red) is expressed at (**A**) 5.5 weeks and (**B**) 10 weeks in culture.

**Supplementary Figure 10. Iba1 expression in organoids.** Iba1 (red) is distributed in the organoids at (**A**) 5.5 weeks, (**B**) 10 weeks and (**C**) 20 weeks in culture.

**Supplementary Figure 11. Changes in gene expression due to hypoxic stress can be protected by minocycline.** (**A**) The expression of cortical marker DCX1 was significantly decreased in ten-day-old organoids grown in 1% oxygen for 72 hours, but not in the presence of 2 uM minocycline. The expression of Yap and Sox10 were also significantly decreased under hypoxic stress, but were mitigated by minocycline (**B, C**). The experiment was repeated 3 separate times and samples from each of the groups were harvested. Each of the samples were then run in triplicate using qPCR. Statistical analysis (2-way ANOVA with multiple comparisons) was performed using Prism software (**** = p < 0.0001, *** = p < 0.001, ** = p < 0.01, * = p < 0.05). GAPDH values are in Supplementary Figure 12C.

**Supplementary Figure 12. GAPDH values for qRT-PCR performed.** (**A**) C_T_ values from GAPDH used to normalize the data presented in Figure 3. (**B**) C_T_ values from GAPDH used to normalize the data presented in Figure 5. (**C**) C_T_ values from GAPDH used to normalize the data presented in Figure 7.

.

**Supplementary Table 1. A list of the human qRT-PCR primers utilized for this study.** DCX1, doublecortin; Eng1, Engrailed homeobox; Foxg1, Forkhead Box G1; GAPDH, Glyceraldehyde-3-Phosphate Dehydrogenase; GFAP, Glial Fibrillary Acidic Protein; Hoxb4, Homeobox B4; Nkx2.1, NK2 Homeobox 1; Olig2, Oligodendrocyte Transcription Factor 2; Satb2, Special AT-Rich sequence-Binding Protein 2; Sox2, SRY (Sex Determining Region Y)-Box 2; Sox10, SRY-Box 10; Tbr1, T-box Brain 1; Yap, Yes Associated Protein 1; Vglut1, Vesicular glutamate transporter 1; F, forward; R, reverse.

Supplementary Table 1.

| DCX1 | F | TTGCCCTGTCTAATTTTGCC |
| --- | --- | --- |
| DCX1 | R | AAAAGGGGCACTTGTGTTTG |
| Eng1 | F | GGACAATGACGTTGAAACGCAGCA |
| Eng1 | R | AAGGTCGTAAGCGGTTTGGCTAGA |
| Foxg1 | F | AGAAGAACGGCAAGTACGAGA |
| Foxg1 | R | TGTTGAGGGACAGATTGTGGC |
| GAPDH | F | ACCACAGTCCATGCCATCAC |
| GAPDH | R | CACCACCCTGTTGCTGTAGCC |
| GFAP | F | AGAGATCCGCACGCAGTATG |
| GFAP | R | TCTGCAAACTTGGAGCGGTA |
| Hoxb4 | F | AAAGCACCCTCTGACTGCCAGATA |
| Hoxb4 | R | ATGGGCACGAAAGATGAGGGAGA |
| Nkx2.1 | F | AACCAAGCGCATCCAATCTCAAGG |
| Nkx2.1 | R | TGTGCCCAGAGTGAAGTTTGGTCT |
| Olig2 | F | CCCTGAGGCTTTTCGGAGCG |
| Olig2 | R | GCGGCTGTTGATCTTGAGACGC |
| Satb2 | F | TAGCCAAAGAATGCCCTCTC |
| Satb2 | R | AAACTCCTGGCACTTGGTTG |
| Sox2 | F | CCCAGCAGACTTCACATGT |
| Sox2 | R | CCTCCCATTTCCCTCGTTTT |
| Sox10 | F | AAGGATTCAGGCTCCGTCCTA |
| Sox10 | R | AGGAAGTGGAAAACCGTGTCC |
| Tbr1 | F | GTCACCGCCTACCAGAACAC |
| Tbr1 | R | ACAGCCGGTGTAGATCGTG |
| Yap | F | GAACCCCAGATGACTTCCTG |
| Yap | R | CTCCTTCCAGTGTTCCAAGG |
| Vglut1 | F | CAGAGTTTTCGGCTTTGCTATTG |
| Vglut1 | R | GCGACTCCGTTCTAAGGGTG |
